# Mycosis fungoides and Sézary syndrome: focus on the current treatment scenario^☆^^☆☆^

**DOI:** 10.1016/j.abd.2020.12.007

**Published:** 2021-05-28

**Authors:** José Antonio Sanches, Jade Cury-Martins, Rodrigo Martins Abreu, Denis Miyashiro, Juliana Pereira

**Affiliations:** a Dermatology Clinic Division, Faculty of Medicine, Hospital das Clínicas, Universidade de São Paulo, São Paulo, SP, Brazil; b Medical Department, Private Institution, São Paulo, SP, Brazil; c Hematology Clinic Division, Faculty of Medicine, Hospital das Clínicas, Universidade de São Paulo, São Paulo, SP, Brazil

**Keywords:** Lymphoma, T-cell, cutaneous, Mycosis fungoides, Sezary syndrome, Therapeutics

## Abstract

Cutaneous T-cell lymphomas are a heterogeneous group of lymphoproliferative disorders, characterized by infiltration of the skin by mature malignant T cells. Mycosis fungoides is the most common form of cutaneous T-cell lymphoma, accounting for more than 60% of cases. Mycosis fungoides in the early-stage is generally an indolent disease, progressing slowly from some patches or plaques to more widespread skin involvement. However, 20% to 25% of patients progress to advanced stages, with the development of skin tumors, extracutaneous spread and poor prognosis. Treatment modalities can be divided into two groups: skin-directed therapies and systemic therapies. Therapies targeting the skin include topical agents, phototherapy and radiotherapy. Systemic therapies include biological response modifiers, immunotherapies and chemotherapeutic agents. For early-stage mycosis fungoides, skin-directed therapies are preferred, to control the disease, improve symptoms and quality of life. When refractory or in advanced-stage disease, systemic treatment is necessary. In this article, the authors present a compilation of current treatment options for mycosis fungoides and Sézary syndrome.

## Introduction

Primary cutaneous lymphomas are a heterogeneous group of non-Hodgkin’s lymphomas that occur on the skin without evidence of extracutaneous disease at the time of diagnosis. They represent 19% of extranodal lymphomas, with an annual incidence in western countries of 1 case per 100,000 inhabitants/year. Primary cutaneous lymphomas are classified by the World Health Organization - European Organization for Cancer Research and Treatment (WHO-EORTC) into Cutaneous T-Cell Lymphomas (CTCL) and Cutaneous B-Cell Lymphomas (CBCL). In the West, CTCLs represent approximately 75% to 80% of all primary cutaneous lymphomas.^1^, ^2^

CTCLs appear primarily on the skin and can progress to lymph nodes, blood and viscera. They are a heterogeneous group of tumors, with considerable variation regarding clinical presentation, as well as their histopathological, immunophenotypic and prognostic characteristics. The updated classification is shown in Table 1, as well as the division according to their agressiveness.^1^

**Table 1:** Classification of T-cell lymphomas with primary cutaneous manifestations according to their clinical behavior, frequency and disease-specific 5-year survival. Based on the WHO-EORTC guidelines.^1^

	Clinical behavior	Frequency (%)	Disease-specific 5-year survival (%)
Mycosis fungoides	Indolent	39	88
Mycosis fungoides variants			
Folliculotropic mycosis fungoides	Indolent	5	75
Pagetoid reticulosis	Indolent	<1	100
Granulomatous slack skin	Indolent	<1	100
Sézary syndrome	Aggressive	2	36
Primary Cutaneous CD30+ Lymphoproliferative Disorders			
Primary cutaneous anaplastic large T-cell lymphoma	Indolent	8	95
Lymphomatoid papulosis	Indolent	12	99
Adult T-cell lymphoma/leukemia	Indolent / Aggressive	<1	NR
Subcutaneous panniculitis-like T-cell lymphoma	Indolent	1	87
Extranodal NK/T-cell lymphoma, nasal type	Aggressive	<1	16
Chronic active EBV infection	Indolent	<1	NR
Primary cutaneous T-cell lymphoma γ/δ	Aggressive	<1	11
Primary cutaneous aggressive epidermotropic CD8+ T-cell lymphoma	Aggressive	<1	31
Primary cutaneous acral CD8+ T-cell lymphoma	Indolent	<1	100
Primary cutaneous CD4+ small/medium T-cell lymphoproliferative disorder	Indolent	6	100
Primary Cutaneous Peripheral T-Cell Lymphoma, NOS	Aggressive	2	15

NR, not reported; NOS, not otherwise specified.

Indolent lymphomas usually have a chronic course with frequent recurrences. The disease is generally considered incurable despite treatment, with a mean survival of 5 to 10 years. However, some patients can survive more than 20 years. Aggressive lymphomas progress faster when compared to indolent forms and can lead to death in a few months.^1^

Mycosis fungoides (MF) is the most common type and accounts for 60% of CTCLs and almost 50% of all primary cutaneous lymphomas. Most patients are adults or elderly, with a male to female ratio of 2:1, and a worldwide incidence of approximately 5–6 cases per million inhabitants/year. Sézary Syndrome (SS) is a rare leukemic variant of CTCL. This article will address the therapeutic possibilities for those two entities.^1^, ^2^

## Mycosis fungoides and Sézary syndrome

MF is a non-Hodgkin’s mature T-cell lymphoma with a primary presentation on the skin, but with potential secondary involvement of lymph nodes, blood and viscera. Skin lesions include patches or plaques that may be localized or disseminated, tumors and erythroderma. The course of MF is variable. In some patients the disease remains limited to the skin for many years, while in others, it may progress more quickly to extracutaneous involvement, with a worse prognosis. SS is an erythrodermic and pruritic form of CTCL, characterized by peripheral lymphadenopathy and the presence of neoplastic T-cells with cerebriform nuclei (Sézary cells), which are clonally related, in the skin, lymph nodes and peripheral blood.^2^

The histopathological analysis typically shows an epidermotropic lymphoma of small to medium-sized lymphocytes with cerebriform nuclei. The neoplastic cells have a mature T-cell phenotype, CD3+, CD4+, CD45RO+, CD8-, with variable loss of CD7 expression.^1^ The CD30 molecule may eventually be expressed in neoplastic cells, but this expression seems to be more frequent and more intense in cases that show transformation to large cell lymphoma. The transformed MF is defined by the presence of large cells (≥ 4-fold the size of a small lymphocyte) surpassing > 25% of the dermal infiltrate or presenting microscopic nodules and is generally associated with a worse prognosis.^3^

The clinical staging of MF and SS is based on the classification of cutaneous (T), lymph node (N), visceral (M) and hematological (B) involvement (Table 2, Table 3). The TNMB classification should include careful examination of the skin (paying attention to the scalp, palms, soles, and perineum), biopsy(ies) of skin lesion(s), complete blood count with screening for Sézary cells, peripheral blood flow cytometry, biochemical tests and imaging studies.^2^ As HTLV-1 is endemic in Brazil, screening for this retrovirus is mandatory for all patients with suspected or proven CTCL, to differentiate MF/SS from adult T-cell lymphoma/leukemia.^4^

**Table 2:** Revised TNMB classification for mycosis fungoides and Sézary syndrome.^2^, ^5^

**Skin (T)**	
T1	Limited patches/plaques (covering <10% of the total skin surface)
T1a	Patches only
T1b	Plaques ± patches
T2	Generalized patches/plaques (covering ≥10% of the total skin surface)
T2a	Patches only
T2b	Plaques ± patches
T3	Tumor(s)
T4	Erythroderma
**Lymph node (N)**	
N0	No clinically abnormal peripheral lymph nodes;
N1	Clinically abnormal peripheral lymph nodes; dermatopathic lymphadenopathy or histopathological involvement by isolated atypical lymphocytes, without alteration of the lymph node architecture
N1a	Negative clone
N1b	Positive clone
N2	Clinically abnormal peripheral lymph nodes; histopathological involvement by aggregates of atypical lymphocytes, without alteration of the lymph node architecture
N2a	Negative clone
N2b	Positive clone
N3	Clinically abnormal peripheral lymph nodes; frank histopathological involvement and partial/complete effacement of the lymph node architecture
NX	Clinically abnormal peripheral lymph nodes; without histopathological confirmation
**Viscera (M)**	
M0	Without visceral involvement
M1	With visceral involvement
**Blood (B)**	
B0	There are no circulating atypical cells (Sézary cells; or <5% of atypical lymphocytes)
B0a	Negative clone
B0b	Positive clone
B1	Low tumor load in the blood (≥5% of lymphocytes are Sézary cells, but not B2 cells)
B1a	Negative clone
B1b	Positive clone
B2	Elevated tumor load in the blood and clonal rearrangement of the TCR (≥1000 Sézary cells/microL. and/or CD4:CD8 ≥ 10, and/or CD4 + CD7 - ≥40%, and/or CD4 + CD26 - ≥30%)

**Table 3:** Clinical staging system for mycosis fungoides and Sézary syndrome.^2^

Clinical staging	TNMB Classification			
Ia	T1	N0	M0	B0 or B1
Ib	T2	N0	M0	B0 or B1
IIa	T1 or T2	N1 or N2	M0	B0 or B1
IIb	T3	N0 to N2	M0	B0 or B1
IIIa	T4	N0 to N2	M0	B0
IIIb	T4	N0 to N2	M0	B1
IVa1	T1 to T4	N0 to N2	M0	B2
IVa2	T1 to T4	N3	M0	B0 to B2
IVb	T1 to T4	N0 to N3	M1	B0 to B2

T, Skin; N, Lymph node; M, Viscera; B, Peripheral blood.

### Treatment options

Most treatments available for MF/SS rarely induce long-term remission. Treatment responses might include the overall response rate (complete remission rate + partial remission rate), complete remission, partial remission (remission of at least 50% of the disease burden), response duration, time until next treatment, progression-free survival, overall survival, disease-specific survival and symptoms and quality of life improvement (Table 4, Table 5). Both treatment and prognosis vary according to the stages of the disease.^6^

**Table 4:** Response rates and duration of responses for skin treatments for MF/SS.

Treatment	Staging	Overall response (%)	Complete response (%)	Response duration	References
Corticosteroid	T1–T2	82–94	25–63	RD: 9 months	9
Nitrogen mustard	T1–T2	58.5–93	13.8–80	–	10, 11
Carmustine	T1–T2	84–98	47–86	–	12
Bexarotene	T1–T2	54–63	10–21	RD: 25 months	13
Imiquimod	T1–T2	80	45	–	14
Resiquimod	T1–T2	75	33	–	15
NB-UVB	T1–T2	–	54–91	RD: 22 months	16
PUVA	T1–T2	–	65–85	RD: 23 months	16
Localized RT	un. MF, 30Gy	100	–	DFS, 5 years: 86%	19
	Ia–III, 9–45Gy	94	88	PFS, 5 years: 85%	17
Total skin irradiation with electrons	T1–T2, 36Gy	95	85–88	PFS, 5 years: 50%	20
	Ia–III, 12Gy	88	27	RD: 70.7 weeks	21

RD, Response Duration; PFS, Progression-Free Survival; DFS, Disease-Free Survival; NB-UVB, narrow-band UVB; RT, Radiotherapy; Gy, Gray; un. MF, unilesional mycosis fungoides.

**Table 5:** Response rates and duration of responses for systemic treatments for MF/SS.

Treatment	Staging	Overall response (%)	Complete response (%)	Response Duration	References
Isotretinoin	MF/ SS	43–80	8–33	RD: 3-15 months	22, 23
Acitretin	Ia–IV	59–64	4–34.6	RD: 28 months	22, 23, 24
Bexarotene	I–IIa	54–67	7–27	RD: 9.9-17.2 months	23, 25
	≥IIb	45–54	2–13		23, 25
Alpha interferon	Ia–IVa	29–80	4–67	RD: 5-8 months	26, 29
Pegylated alpha-interferon	Ib–III	50–83	33–67	–	28
Pegylated liposomal doxorubicin	Ib–IV	41–88	6	RD: 6-13.3 months	32, 33
Gemcitabine	T3–T4	47–70	11.5–22	RD: 10-15 months	34, 38
Chlorambucil	SS	85	35	RD: 16.5 months	36
	III–IVb	100	53.8		35
Methotrexate, low dose	T2	33	33	RD: 15 months	39
	III	56	41	RD: 31 months	39
Pralatrexate	IIa–IV	40.8	6.1	RD: 6 months	40
Romidepsin	IIb	34–38	6–7	RD: 15 months	41
Vorinostat	Ib–IVa	30	–	RD: 5 months	42
Alemtuzumab	adv. MF–SS	38–84	0–47	RD: 4 months	44, 45
Brentuximab vedotin	Ia–IVb	50	10	TNT: 13.4 months	46
Mogamulizumab	Ia–IVb/SS	28 (MF: 21; SS: 37)	-	PFS: 7.7 months	47
CHOP	IIb	66	–	TNT: 5.7 months	38
EPOCH	IIb–IV	80	–	PFS: 8 months	38
Fludarabine + CFM	IIb–III	55	–	RD: 10 months	38
Fludarabine + IFN	IIa–IVb	40–58	–	PFS: 5.9 months	38
IFN + PUVA	Ia–IVa	80.6	75	RD: 32 months	30
		–	–	PFS, 5 years: 75%	30
	Ia–IIa	98	84	PFS, 5 years: 27%	31
IFN peg + PUVA	Ia–IV	88	44	PFS: 30.9 months	27
IFN + retinoid	Ia–IIb	38	6	–	29
Bexaroteno + PUVA	Ib–IIa	77	31	RD: 5.8 months	52
Isolated ECP	III–IVa1	24	9	TNT: 14 months	49, 50, 51
ECP + IFN	III–IVa1	45.4	18.2	–	49, 50
ECP + IFN + Bexarotene	III–IVa1	88.2	32.4	RD: 4 months	49, 50

PFS, Progression-Free Survival; RD, Response Duration; TNT, Time to Next Treatment; adv. MF, Advanced Mycosis Fungoides; SS, Sézary syndrome;; CHOP, Cyclophosphamide, Doxorubicin, Vincristine, and Prednisolone; EPOCH, Etoposide, Vincristine, Doxorubicin, Cyclophosphamide, and Prednisolone; ECP, Extracorporeal Photopheresis; CFM, Cyclophosphamide; IFN, Interferon; peg. IFN, pegylated Interferon.

The treatment modalities can be divided into two groups: skin-directed therapies and systemic therapies. The treatments can be done as monotherapy or combined therapy. Skin-directed therapies include topical agents, phototherapy and radiotherapy.^7^ Systemic therapies include biological response modifiers, immunotherapies and chemotherapeutic agents.^8^ For early-stage MF (stage Ia – IIa), skin-directed therapies are the treatment of choice, aiming at controlling the disease, improving symptoms and quality of life. In refractory early-stage MF, late-stage MF (stage ≥ IIb) and SS, systemic treatment, combined or not with skin-directed therapy, is required. Treatment modalities are reviewed in this article.

The approval and availability of drugs in the United States, Europe and Brazil are described in Table 6, Table 7. Extracorporeal photopheresis, narrow-band UVB phototherapy, psoralen-UVA (PUVA) photochemotherapy, and radiotherapy are more widely available.

**Table 6:** Approval of topical treatments for cutaneous T-cell lymphoma in the United States, Europe and Brazil, according to the package insert registered in the local regulatory agencies (FDA, EMA and ANVISA), respectively.

Treatment	Class of Medication	United States of America (FDA)	Europe (EMA)	Brazil (ANVISA)
Corticosteroid	Corticosteroid	Available.	Available.	Available.
		Approved, but does not have a package insert indication for CTCL.	Approved, but does not have a package insert indication for CTCL.	Approved, but does not have a package insert indication for CTCL.
Mechlorethamine	Alkylating agent	Available.	Available.	Not available.
		Approved for topical treatment of MF Ia and Ib in patients who previously received some type of skin therapy.	Approved for topical treatment of CTCL.	Not approved.
Carmustine	Alkylating agent	Available for intravenous therapy.	Available for intravenous therapy.	Available for intravenous therapy.
		Approved, but does not have a package insert indication for CTCL.	Approved, but does not have a package insert indication for CTCL.	Approved, but does not have a package insert indication for CTCL.
Bexarotene	Retinoid	Available.	Not available.	Not available.
		Approved for skin lesions in patients with CTCL Ia and Ib with refractory or persistent disease after other therapies or those who do not tolerate other therapies.	Not approved.	Not approved.
Imiquimod	Immune response modifier	Available.	Available.	Available.
		Approved, but does not have a package insert indication for CTCL.	Approved, but does not have a package insert indication for CTCL.	Approved, but does not have a package insert indication for CTCL.
Resiquimod	Immune response modifier	Not available.	Available.	Not available.
		Not approved.	Sole designation for the treatment of CTCL.	Not approved.

FDA, Food and Drug Administration (https://www.fda.gov/drugs); EMA, European Medical Agency (https://www.ema.europa.eu/en); ANVISA, National Health Surveillance Agency (*Agência Nacional de Vigilância Sanitária*; http://portal.anvisa.gov.br/); CTCL, Cutaneous T-Cell Lymphoma; MF, Mycosis Fungoides.

**Table 7:** Approval of systemic treatments for cutaneous T-cell lymphomas in the United States, Europe and Brazil, according to the package insert registered at the local regulatory agencies (FDA, EMA and ANVISA), respectively.

Treatment	Class of medication	United States of America (FDA)	Europe (EMA)	Brazil (ANVISA)
Isotretinoin	Retinoid	Available.	Available.	Available.
		Approved, but does not have a package insert indication for CTCL.	Approved, but does not have a package insert indication for CTCL.	Approved, but does not have a package insert indication for CTCL.
Acitretin	Retinoid	Available.	Available.	Available.
		Approved, but does not have a package insert indication for CTCL.	Approved, but does not have a package insert indication for CTCL.	Approved, but does not have a package insert indication for CTCL.
Bexarotene	Retinoid	Available.	Available.	Not available.
		Approved for cutaneous manifestations of CTCL in patients refractory to at least one previous systemic therapy.	Approved for cutaneous manifestations of CTCL in patients refractory to at least one previous systemic therapy.	Not approved.
Alpha-interferon	Immunomodulator	Available.	Available.	Available.
		Approved, but does not have a package insert indication for CTCL.	Approved, but does not have a package insert indication for CTCL.	Approved, but does not have a package insert indication for CTCL.
Pegylated liposomal doxorubicin	Anthracycline chemotherapeutic drug	Available.	Available.	Available.
		Approved, but does not have a package insert indication for CTCL.	Approved, but does not have a package insert indication for CTCL.	Approved, but does not have a package insert indication for CTCL.
Gemcitabine	Pyrimidine antagonist chemotherapeutic drug	Available.	Available.	Available.
		Approved, but does not have a package insert indication for CTCL.	Approved, but does not have a package insert indication for CTCL.	Approved, but does not have a package insert indication for CTCL.
Chlorambucil	Alkylating chemotherapeutic drug	Available.	Not available.	Available.
		Approved, but does not have a package insert indication for CTCL.	Not approved.	Approved for non-Hodgkin's lymphomas.
Methotrexate	Antimetabolite	Available.	Available.	Available.
		Approved for advanced MF.	Approved, but does not have a package insert indication for CTCL.	Approved for non-Hodgkin's lymphomas.
Pralatrexate	Antimetabolite	Available.	Not available.	Not available.
		Approved for recurrent or refractory peripheral T-cell lymphomas.	Not approved.	Not approved.
Romidepsin	HDAC inhibitor	Available.	Not available.	Not available.
		Approved for patients with CTCL who received at least one previous systemic therapy.	Not approved.	Not approved.
Vorinostat	HDAC inhibitor	Available.	Not available.	Not available.
		Approved for patients with CTCL with progressive, persistent, or recurrent disease after two systemic therapies.	Not approved.	Not approved.
Brentuximab vedotin	Antibody-drug conjugate	Available.	Available.	Available.
		Approved for patients with MF or PCALCL expressing CD30 who received previous systemic treatment.	Approved for patients with CD30-positive CTCL who received at least one previous treatment.	Approved for PCALCL or patients expressing CD30 who received previous systemic treatment
Alemtuzumab	Monoclonal antibody	Available.	Available.	Available.
		Approved, but does not have a package insert indication for CTCL.	Approved, but does not have a package insert indication for CTCL.	Approved, but does not have a package insert indication for CTCL.
Mogamulizumab	Monoclonal antibody	Available.	Available.	Not available.
		Approved for patients with relapsing or refractory MF or SS after at least one previous systemic therapy.	Approved for patients with MF or SS after at least one previous systemic therapy.	Not approved.

FDA, Food and Drug Administration (https://www.fda.gov/drugs); EMA, European Medical Agency (https://www.ema.europa.eu/en); ANVISA, National Health Surveillance Agency (*Agência Nacional de Vigilância Sanitária*; http://portal.anvisa.gov.br/); CTCL, Cutaneous T-Cell Lymphomas; MF, Mycosis Fungoides; HDAC, histone deacetylase; PCALCL, Primary Cutaneous Anaplastic Large Cell Lymphoma; SS, Sézary Syndrome.

#### Skin-directed therapies

##### Topical drug therapies

###### Corticosteroids

Topical corticosteroids are the most widely used anti-inflammatory agents in dermatology. They inhibit the binding of lymphocytes to the endothelium, inhibit intercellular adhesion and induce apoptosis of neoplastic lymphoid cells. They are often formulated as cream, ointment, lotion or gel. According to the topical corticosteroid and the formulation, the drug potency can be stratified into seven classes: class I (super-high potency, e.g., 0.05% clobetasol propionate cream); class II (high potency, e.g., 0.05% betamethasone dipropionate ointment; class III (medium-high potency, e.g., 0.05% betamethasone dipropionate cream); class IV (medium potency, e.g., 0.1% triamcinolone acetonide cream); class V (lower-medium potency, e.g., 0.1% betamethasone valerate cream), class VI (low potency, e.g., 0.05% desonide cream) and class VII (lowest potency, e.g., 1% hydrocortisone acetate cream). Topical steroids are applied once or twice a day, for weeks to months, until complete regression or considerable improvement of the lesions is achieved. Prolonged use of topical corticosteroids can result in skin atrophy, striae and mild skin irritation. Systemic absorption with the use of high-potency corticosteroids applied to large surfaces of the skin, causing clinically evident adrenal insufficiency and Cushing's syndrome, are rarely observed.^7^, ^9^

###### Mechlorethamine

Mechlorethamine, chlormethine or nitrogen mustard, is an alkylating chemotherapeutic agent that affects rapidly-dividing cells, acting as a cytotoxic agent in DNA. Nitrogenous mustard was originally available as a lyophilized powder. The powder could then be used in a cream or aqueous solution. The cream was prepared at the initial concentration of 10 to 20 mg of nitrogen mustard per 100 g of petrolatum or similar. Regarding the aqueous preparation, the patients prepared the solution at a concentration of 10 to 20 mg per 100 mL of water. The preparations were applied once a day on specific lesions or the total skin surface (depending on the T-classification), except in the genital area, for weeks to months. It is currently available in the United States and Europe as a 0.02% gel formulation for more localized use. Short-term adverse events include pruritus, burning sensation and irritant or allergic contact dermatitis. In the long term, skin hyper- or hypopigmentation and a slight increase in the risk of non-melanoma skin cancer can be observed, especially when combined with other therapies, such as phototherapy. The topical use of this substance does not cause myelosuppression.^7^, ^10^, ^11^

###### Carmustine

Carmustine, also known as bischlorethyl nitrosourea (BCNU), is an alkylating agent that forms cross-links in DNA, preventing DNA replication and transcription. To prepare the alcoholic solution, 100 mg of powdered carmustine is dissolved in 5 mL of 95% ethanol. This 5 mL solution is then placed in a glass container and diluted with an additional 50 mL of 95% ethanol. This results in a concentration of 2 mg / mL (0.2%), called stock solution. For total body use, 5 mL (10 mg of BCNU) of the stock solution is diluted in 60 mL of water. It is applied once a day. For localized lesions, the solution volume is adjusted to the affected skin area. For extremely limited disease (< 3% of skin affected), applications can be done with the undiluted stock solution. Topical carmustine can also be incorporated into a petrolatum ointment at a concentration of 10 mg/100 g of petrolatum. Usually, the maximum daily dose is 10 mg/day, with a treatment duration of six to twelve weeks. This treatment is usually not recommended for patients with skin involvement >3%, as systemic absorption can lead to hematological toxicity including leukopenia, thrombocytopenia and anemia. Local reactions are commonly observed, and most patients develop erythema and a burning sensation, especially in the skin folds.^7^, ^12^

###### Bexarotene

Bexarotene is a retinoid (derived from vitamin A) that selectively activates retinoid X receptors, inducing cell differentiation and apoptosis. Topical bexarotene is available in a 1% formulation. Bexarotene is applied to the lesions every night during the first week of treatment and then twice a day. The use of this medication is generally limited to patients with less than 15% of the body surface affected. The most common adverse effects are mild to moderate irritant dermatitis, pruritus and burning sensation at the application site.^7^, ^13^

###### Imiquimod and resiquimod

Imiquimod is a toll-like receptor 7 agonist that increases interferon production. Interferon leads to antiviral, antiproliferative and antiangiogenic effects. It also stimulates Langerhans cells to migrate to the lymph nodes, activating T lymphocytes. Topical imiquimod is available as a 5% cream and is applied to the lesions, three to five times a week, until their resolution. The most often reported toxicity is a local inflammatory reaction, with erythema, edema, vesicles, and ulceration/erosion. These signs reflect the immune system activation and, if no inflammatory response is observed, an adequate response to therapy is less likely to occur. Some patients may experience systemic symptoms (e.g., flu-like symptoms).^7^, ^14^ Resiquimod is an agonist of toll-like receptors 7 and 8. Toll-like receptor 8 is expressed by dendritic cells derived from myeloid cells and these are strongly activated by the drug. Similar to imiquimod, as a toll-like receptor 7 agonist, interferon production is increased. The drug, in a 0.06% or 0.03% gel, is applied to the skin lesions, three times a week, for eight weeks. There is less skin irritation compared to imiquimod, with erythema and superficial erosions. Low fever may occur for a short period. No serious adverse events have been described.^7^, ^15^

##### Radiation therapies

###### Phototherapy

This is a physical therapy based on ultraviolet (UV) light. The UV light acts by stopping the cell cycle or apoptosis of keratinocytes, Langerhans cells and lymphocytes and by selectively decreasing the production of pro-inflammatory cytokines by T-cells. There are different treatment modalities based on the wavelength and its association or not with a psoralen (photosensitizing agent). The most widely used are narrow-band UVB phototherapy (NB-UVB) and PUVA (psoralen + ultraviolet A), also known as photochemotherapy. It is an important treatment option for patients with early-stage MF or as adjunctive therapy for more advanced stages. The treatment dose and duration vary between institutions, as well as the use or not of maintenance therapy after the lesions improve (being less frequently used to reduce the total accumulated dose of UV).^7^, ^16^

####### Narrow-band UVB

Phototherapy with NB-UVB comprises a wavelength from 311 to 313 nm. Due to its shorter wavelength, it is absorbed mainly in the epidermis with less capacity to penetrate more deeply into the skin when compared to UVA. Therefore, its main direct effects are on epidermal keratinocytes, Langerhans cells, follicular infundibulum, and upper dermal cells, including lymphocytes. It is more frequently used when the disease is in the stage of non-infiltrated lesions (patches) (T1a and T2a). It has advantages over PUVA because it does not require the use of a photosensitizing agent (psoralen), with a lower risk of skin cancer (with cumulative doses) and can be used during pregnancy. The treatment is administered on an outpatient basis, three to five times a week, with a gradual increase in the dose. The most common acute adverse events (within 24 hours) are erythema, pruritus, burning sensation, blistering, tanning, and xerosis. Regarding skin neoplasms, a literature review published in 2005 that included more than 3,400 patients (mainly patients with psoriasis) treated with broadband UVB (BB-UVB) or NB-UVB, did not show an increase in the risk of skin cancer, except for those treated with UVB and PUVA. Chronic exposure to UV light is associated with the formation of cataracts and ocular pterygium. The use of eye protection during phototherapy with UVB is indicated to prevent this risk. However, for those who need periocular treatment, it is safe to undergo the sessions with their eyes closed, since there is only a negligible transmission of UV light through the eyelids.^7^, ^16^

####### PUVA photochemotherapy

In this treatment modality, photosensitizing agents (psoralens) are used orally or topically before exposure to UVA (wavelength from 320 to 400 nm). UVA (especially UVA1 - 340 at 400 nm) can penetrate the entire dermis, affecting lymphocytes in the deep dermis, fibroblasts, dermal dendritic cells, mast cells, endothelial cells, macrophages, and deeper parts of the hair follicle. It is a better option for the treatment of more infiltrated lesions in plaques and folliculotropic MF. PUVA treatment is administered three times a week until lesion regression is achieved. Eye protection should be worn not only during sessions, but for 24 hours after ingesting the psoralen. It should be noted that cumulative UV doses are associated with an increased risk of associated skin malignancies, mainly squamous cell carcinoma, but also basal cell carcinoma. There are still controversies regarding the increased risk for melanoma. Oral psoralen can cause nausea and, rarely, hepatotoxicity. Psoralens are absorbed by the lens, although they disperse within 24 hours without exposure to UVA. In the presence of UVA, 8-MOP binds to nucleic acids and proteins in the lens and remains for long periods, theoretically increasing the risk of cataracts. Therefore, it is recommended to use UVA eye protection during exposure to sunlight, for 24 hours after ingesting the psoralen.^7^, ^16^

###### Radiotherapy

Radiotherapy (RT) involves the use of ionizing radiation through photons or electrons to destroy neoplastic cells. Ionizing radiation acts by causing damage to the neoplastic tissue DNA, leading to cell death. Mycosis fungoides is a very radiosensitive tumor and radiotherapy is a very effective treatment modality.^7^

####### Local radiotherapy

For individual or localized lesions, conventional photon radiotherapy or electron beam radiotherapy is used.^6^, ^7^ Low-dose regimens with 8 Gy in two fractions are effective for plaques or small tumors. For the treatment of large areas, smaller doses per fraction (20–30 Gy in 10 to 15 fractions) should be considered. The clinical target volume of radiotherapy is defined with a margin of at least 1 cm around the lesion. Peripheral nodal disease and visceral disease can also be treated with localized RT. MF is a multifocal disease, and local control with radiotherapy is frequently used as a palliative approach. Therefore, it is recommended to use the minimum dose of radiotherapy to attain local control, which allows retreatments or treatments in adjacent areas. The adverse event observed in localized radiotherapy is radiodermatitis, and the radiodermatitis recall phenomenon may occur in patients treated with chemotherapy.^17^, ^18^, ^19^

####### Total skin electron beam therapy

Total skin electron beam therapy (TSEBT) is a special technique that allows homogeneous irradiation of the entire skin surface. The usual total dose for treatment is 36 Gy, administered as one to two Gy per session for five to nine weeks.^20^ Complete remission rates are high; however, recurrences are frequent. Low-dose TSEBT is performed with 12 Gy, one Gy per fraction for three weeks.^21^ Consensus guidelines on the use of TSEBT in MF have been published.^18^ Treatment-related toxicity depends on the radiation dose used and the tumor location. Adverse events include erythema, hair loss, temporary nail growth arrest, hand and foot edema, minor nasal bleeding, blisters on the fingers and toes, anhidrosis, mild parotitis, gynecomastia in men, keratitis due to the use of internal eye shields, permanent nail dystrophy, xerosis, permanent alopecia, dysesthesia in the fingers.^7^ Low-dose TSEBT is better tolerated and adverse events are transient and milder when compared to the standard dose. However, the response rates are lower.^21^

#### Systemic therapy

##### Retinoids: isotretinoin, acitretin and bexarotene

Retinoids are natural and synthetic analogs of vitamin A that bind to several classes of proteins, including retinoid-binding proteins and nuclear retinoid receptors. They lead to the activation of DNA regulatory regions involved in the regulation of cell growth, differentiation and apoptosis. The nuclear retinoid receptors belong to two families: Retinoic acid receptors (RARs) and retinoid X receptors (RXRs). Isotretinoin is a first-generation non-aromatic retinoid. It was the first retinoid used for the off-label treatment of CTCL. It is used in daily oral doses of 0.2 to 1.0 mg/kg. Adverse events include dry skin and mucous membranes, the elevation of blood lipids and teratogenicity (pregnancy should be avoided for one month after treatment discontinuation).^22^ Acitretin is a second-generation monoaromatic retinoid. Their structure makes them more lipophilic with greater bioavailability than the first-generation retinoids. It is used in daily oral doses of 0.3–0.5 mg/kg. The safety profile is similar to that of isotretinoin, but pregnancy should be avoided for two to three years after treatment discontinuation.^22^ Bexarotene is a third-generation polyaromatic retinoid that is highly selective for the RXR receptor. It is administered at a daily oral dose of 300 mg/m^2^. The safety profile is similar to that of isotretinoin and acitretin, but it has additional adverse events, such as central hypothyroidism and hypertriglyceridemia in most patients. Pregnancy should be avoided for one month after treatment discontinuation.^8^, ^23^, ^24^, ^25^

##### Interferon

Interferon (IFN) is a Th1 cytokine. There are three types of recombinant interferons (alpha, beta, gamma) currently available for therapeutic use. IFN-α can be found in its pegylated form. All recombinant IFNs are active in the treatment of MF/SS, but IFN-α is the most frequently studied. IFN-α regulates the cell cycle, promotes the suppression of oncogenes and modulates cell adhesion.^26^ IFN-γ is more frequently studied and used in Japan. Treatment with IFN-α is usually started with a dose of 3 million units via subcutaneous route, three times a week. The dose can be increased up to 9 million units per application. Pegylated IFN-α, a formulation in which the drug is encapsulated in liposomes, resulting in increased half-life and better accumulation in tumor tissues, is administered via subcutaneous route, at a dose of 1.5 μg/kg, once a week.^27^, ^28^ IFN-γ is administered subcutaneously at a dose of 50 μg/m^2^ for patients with a body surface area >0.5 m^2^ and 1.5 μg/kg/dose for patients with a body surface area ≤ 0.5 m^2^. It is used daily or three times a week.^29^ Adverse events from interferons are dose-dependent and include flu-like symptoms, thyroid dysfunction, increased transaminases, leukopenia, thrombocytopenia, depression, and arrhythmias.^29^, ^30^, ^31^

##### Pegylated liposomal doxorubicin

Doxorubicin is an anthracycline cytotoxic agent. It binds to nucleic acids, inhibiting the progression of topoisomerase II, interrupting the DNA replication process. Pegylated liposomal doxorubicin is encapsulated in liposomes, resulting in increased half-life and better accumulation in tumor tissues. Pegylated liposomal doxorubicin is administered intravenously, at a dose of 20 mg/m^2^ on days 1 and 15, every 28 days. The main adverse events are usually mild or moderate and include anemia, asthenia, nausea, vomiting, and palmoplantar erythrodysesthesia.^8^, ^32^, ^33^

##### Gemcitabine

Gemcitabine (2',2'-difluorodeoxycytidine) is a classic chemotherapeutic agent from the family of nucleoside analogs. It acts by blocking the formation of new DNA, resulting in cell death. It is administered intravenously, in doses of 1200 mg/m^2^ on days 1, 8, and 15 of a 28-day regimen. Treatment is well tolerated and the main adverse event is hematological toxicity, which is generally mild.^8^, ^34^

##### Chlorambucil

Chlorambucil is an alkylating agent and forms cross-links in DNA, causing DNA damage and affecting replication and transcription. Chlorambucil can be used orally in a continuous regimen with 2–6 mg/day of chlorambucil plus 20 mg/d of prednisone, or in pulses of 10 to 12 mg/day of chlorambucil for 3 days and fluocortolone, with a dose of 75 mg on the first day, 50 mg on the second day and 25 mg on the third day, every two weeks.^35^, ^36^ Doses and intervals can be reduced and prolonged as the disease improves. The continuous and prolonged use of chlorambucil increases the risk of myelosuppression and the development of leukemia, especially acute myeloid leukemia.

##### Combined chemotherapy

The most frequently used combinations are Cyclophosphamide, Doxorubicin, Vincristine, and Prednisone (CHOP); Cyclophosphamide, Vincristine, and Prednisone (CVP); Cyclophosphamide, Doxorubicin, Vincristine, and Etoposide (CHOEP); CVP plus Methotrexate (MTX). Combined chemotherapy regimens are associated with high response rates (70%–80%), but often of short duration (around 4 months). Multidrug chemotherapy is also associated with myelosuppression and infectious complications. Therefore, with rare exceptions, the sequential administration of a single chemotherapeutic drug is the strategy of choice.^37^, ^38^

##### Antifolate drugs: methotrexate and pralatrexate

MTX is an antifolate-type antimetabolite. It competitively inhibits dihydrofolate reductase and, consequently, the folic acid metabolism, acting by inhibiting the synthesis of DNA, RNA, thymidylates and proteins. Low-dose MTX has been used to treat early-stage MF and SS for many years.^39^ MTX is used in a dose of 10 to 25 mg, once a week, orally or subcutaneously. The adverse effects include gastrointestinal symptoms (nausea, vomiting, stomatitis, diarrhea), anemia, leukopenia, thrombocytopenia, increased transaminases, liver fibrosis, and pneumonitis.^8^, ^40^ Pralatrexate is also a folate analog with activity demonstrated in patients with MF/SS. It is administered intravenously in a dose of 15 mg / m^2^ per week for three weeks, in four-week cycles. Adverse events are similar to those seen with MTX, but they tend to be more common and severe. The most common side effects include mucositis, fatigue, nausea, vomiting, anorexia, skin toxicity, epistaxis, and anemia.^8^, ^40^

##### Histone deacetylase inhibitors: romidepsin and vorinostat

Histone Deacetylase (HDAC) is a class of enzymes responsible for catalyzing the removal of acetyl groups from histones, being crucial modulators of transcriptional epigenetic regulation. Two drugs from this class have FDA approval for use in CTCL, mainly in refractory cases: romidepsin and vorinostat.^41^, ^42^ Romidepsin is administered as a single agent in a dose of 14 mg/m^2^ intravenously on days 1, 8 and 15 of a 28-day cycle. The most common related adverse events are nausea and fatigue.^43^ Vorinostat is an oral HDAC inhibitor. It is administered at a daily dose of 400 mg per day. If not tolerated, it can be reduced to 300 mg/day or 300 mg, five days a week. The most common adverse events are diarrhea, fatigue, nausea, and anorexia.^43^

##### Alemtuzumab

Alemtuzumab is a humanized anti-CD52 monoclonal antibody. CD52 is a surface glycoprotein present in T and B lymphocytes, monocytes and macrophages. Alemtuzumab binds to CD52, causing destruction of neoplastic cells by antibody-dependent cell cytotoxicity and complement fixation. Response rates are higher in SS than in MF, because alemtuzumab leads to the depletion of central memory T-cells in the blood and skin of patients with SS. On the other hand, the drug does not affect memory/effector T-cells residing on the skin in MF. Recent evidence suggests that alemtuzumab is not effective in tumoral or transformed MF. The standard dose is 30 mg administered intravenously, three times a week, for up to 12 weeks.^8^, ^44^ Subcutaneous alemtuzumab can also be used in a low-dose regimen of 10 to 15 mg every other day, 3 times a week.^45^ Infusion reactions (fever, nausea, hypotension, fatigue, rash, hives, bronchospasm) are seen in more than half of the patients. Cytopenias (lymphopenia, neutropenia, anemia, thrombocytopenia) are observed in almost all patients. Severe infections (cytomegalovirus, generalized herpes simplex, fatal aspergillosis, and mycobacterial pneumonia), can be observed, especially in patients heavily pretreated.^8^ The low-dose regimen can reduce infectious complications in patients with MF.^45^ The treatment with alemtuzumab requires antibiotic and antiviral prophylaxis, as well as careful observation for the development of cardiac infection and toxicity.^8^

##### Brentuximab vedotin

Brentuximab vedotin (BV) is an antibody-drug conjugate directed to the CD30 molecule. CD30 belongs to the tumor necrosis factor receptor superfamily. The drug in the antibody-drug conjugate is monomethyl auristatin E (MMAE), an anti-tubulin agent. In normal tissues, CD30 has an expression profile that is rather restricted to activated T, B and NK/T-cells, but is highly expressed in Reed Sternberg cells of Hodgkin's lymphoma, in anaplastic large-cell lymphoma and other non-Hodgkin lymphoma subtypes, for instance, in primary cutaneous anaplastic large-cell lymphoma (pcALCL) and certain cases of MF. This expression profile makes CD30 an ideal target for therapies based on monoclonal antibodies. After the binding of BV to CD30 on the surface of neoplastic cells and their internalization, MMAE release occurs. MMAE will then exert its potent cytostatic effect, inhibiting the assemblage of the microtubules, inducing the cell cycle to stop, and resulting in death due to apoptosis of tumor cells. The safety and efficacy of BV were evaluated in the first phase 3 study against a standard therapy (MTX or bexarotene) for CTCL, demonstrating high rates of durable and clinically significant responses. The typical starting dose is 1.8 mg/kg given intravenously every three weeks. The main toxicities are peripheral neuropathy, neutropenia, fatigue, nausea, and alopecia.^46^

##### Mogamulizumab

Mogamulizumab is a humanized monoclonal antibody that targets the C-C chemokine receptor type 4 (CCR4) expressed on neoplastic cells. CCR4 is expressed on the surface of tumor cells of most patients with adult T-cell lymphoma/leukemia (ATLL) and is selectively expressed in other subtypes of peripheral T-cell lymphoma and cutaneous T-cell lymphoma. After binding to its target, mogamulizumab acts through antibody-dependent cell-mediated cytotoxicity to destroy tumor cells. Mogamulizumab is administered intravenously in a dose of 1.0 mg/kg weekly, for four weeks, followed by a dose every two weeks until disease progression. Infusion-related and drug-related rashes are common; other toxicities include diarrhea, nausea, thrombocytopenia, dysgeusia, and elevated serum creatinine levels.^47^

##### Extracorporeal photopheresis

Extracorporeal photopheresis (ECP) is an immunomodulatory therapy in which leukapheresis removes the patient's leukocytes, which are then treated with 8-methoxypsoralen and UVA and reinfused in the patient. It is the first-line treatment for SS and erythrodermic MF.^6^, ^48^ Each cycle of ECP is generally administered on two consecutive days every two to four weeks for at least six months. With the development of 8-MOP in solution, ECP can be administered directly into the apheresis bag, and the patient is not exposed to the systemic drug absorption, minimizing adverse gastrointestinal effects and systemic photosensitivity. Sporadic side effects include headache, pyrexia, myalgia, mild anemia, thrombocytopenia, and photophobia. Hypotension, vasovagal syncope, infection at the injection site, worsening of skin lesions and sepsis, usually due to central venous access, are rare. There are no reports of opportunistic infections or malignancies, as ECP is not an immunosuppressive treatment.^48^, ^49^, ^50^, ^51^

##### Combined therapies

The combination of treatments is a well-established strategy to increase therapeutic efficacy in treatments for MF. It includes combining skin-directed therapies with systemic ones, or two or more systemic therapies. The most widely used combinations are retinoids and PUVA (RE-PUVA), interferon, and PUVA or ECP with interferon and/or bexarotene.^49^, ^52^

##### Hematopoietic stem cell transplantation (HSCT)

Allogeneic stem cell transplantation (allo-HSCT) can induce lasting remissions and is the only treatment with curative intent. Due to the high morbidity and mortality rates associated with allo-HSCT, good patient selection is essential for a successful treatment. Careful counseling is necessary and the indication should focus mainly on younger patients with good performance, with advanced stages of the disease and low tumor burden at the time of transplantation and who are aware of the high risk of progression and poor prognosis.^53^

### Response rates and treatment recommendations

The response rates and duration with the different skin-directed treatments, systemic treatments and combinations of treatments reported in the literature are shown in Table 4, Table 5. It is important to note that the studies are methodologically heterogeneous and many are not controlled. The summary of the treatment recommendations of the Cutaneous Lymphoma Group of the Dermatology and Hematology Divisions of HC-FMUSP can be seen in Table 8. The recommendations take into account the clinical experience and the best scientific evidence for treatments at different stages of the disease. In refractory early disease and advanced disease, despite the use of systemic therapies (biological response modifiers and/or chemotherapy), the association of skin-directed therapies is frequent. The availability and/or approval in Brazil of the described treatments do not guarantee patient access to the medications, both in the public and private sectors. Several factors can influence access to treatments, such as institutional clinical protocols, state guidelines, supplementary health treatment protocols, particularities of health plans/health insurance, opinions of medication standardization commissions, or even individual requests for medications, made by the patients. There are drugs, both for skin-directed treatments and systemic treatments, which are of crucial importance in the treatment of MF and SS, not yet available in Brazil. Thus, it is suggested that the recommendations be evaluated and adapted to the reality of each service. The authors believe that the development of a National Clinical Protocol for the Approach of Cutaneous Lymphomas is fundamental to guarantee universal access, for all patients, to the different treatments available in the country. Unquestionably, the best care given to cancer patients translates into a reduction in the physical and emotional impacts related to the disease itself and the individual, as well as in a significant reduction in the associated economic costs, both direct and indirect ones.

**Table 8:** Recommendations of the Cutaneous Lymphomas Group of the Dermatology and Hematology Divisions of HC-FMUSP for the treatment of mycosis fungoides and Sézary syndrome.

CS	First-line	Second line	Third line
Ia	Topical corticosteroids	Localized topic PUVA	Localized radiotherapy (if regionalized disease)
	Phototherapy with NB-UVB	PUVA	
	Topical mechlorethamine^a^		
	Topical carmustine		
	Bexarotene gel^a^		
	Imiquimod / Resiquimod		
Ib, IIa	Phototherapy with NB-UVB	Systemic retinoids^b^ ± phototherapy with NB-UVB or PUVA	Total skin electron beam radiotherapy
	PUVA phototherapy	Alpha interferon ± phototherapy with NB-UVB or PUVA	Low-dose methotrexate
IIb	Systemic retinoids ± phototherapy with NB-UVB or PUVA ± localized RT	Total skin electron beam radiotherapy	Monochemotherapy (gemcitabine, pegylated liposomal doxorubicin)
		Low-dose methotrexate	
		Brentuximab vedotin (if CD30-positive)	HDACi^a^
	Alpha-interferon ± phototherapy with NB-UVB or PUVA ± localized RT		Mogamulizumab
			Polychemotherapy (CHOP type)
			Allogeneic hematopoietic stem cell transplantation
IIIa, IIIb	Systemic retinoids^b^ ± PUVA	Chlorambucil + prednisone	HDACi^a^
	Alpha interferon ± PUVA	Low-dose methotrexate	Polychemotherapy (CHOP type)
	ECP ± systemic retinoids^b^ ± alpha-interferon	Monochemotherapy (gemcitabine, pegylated liposomal doxorubicin)	Allogeneic hematopoietic stem cell transplantation
IVa1, SS	ECP ± alpha-interferon ± systemic retinoids^b^	Chlorambucil + prednisone	Monochemotherapy
		Low-dose methotrexate	Polychemotherapy (CHOP type)
	Alpha interferon ± PUVA	Alemtuzumab	Allogeneic hematopoietic stem cell transplantation
		Mogamulizumab^a^	
IVa2	Monochemotherapy (gemcitabine, pegylated liposomal doxorubicin)	Brentuximab vedotin (if CD30-positive)	HDACi^a^
			Polychemotherapy (CHOP type)
	Radiotherapy if localized nodal disease		Allogeneic hematopoietic stem cell transplantation
IVb	Monochemotherapy (gemcitabine, pegylated liposomal doxorubicin)	Brentuximab vedotin (if CD30-positive)	Polychemotherapy (CHOP type)
			Allogeneic hematopoietic stem cell transplantation

HC-FMUSP, Hospital de Clínicas, Faculdade de Medicina, Universidade de São Paulo; CS, clinical-stage; PUVA, psoralen + ultraviolet A; NB-UVB, narrow-band UVB; RT, radiotherapy; HDACi, histone deacetylase inhibitors; CHOP, cyclophosphamide, doxorubicin, vincristine, and prednisolone; SS, Sézary syndrome; ECP, extracorporeal photopheresis.

a Agent not available in Brazil.

b Bexarotene is not available in Brazil, being replaced by acitretin and, less commonly, by isotretinoin.

## Conclusion

As most of the available treatments for MF and SS rarely induce long periods of remission or complete cure, the main treatment objectives are to control the disease symptoms, improve patient quality of life, prolong progression-free survival and overall survival. Also, as an indolent disease, with a 5-year disease-related survival of approximately 90% in most cases, and with the importance of the tumor microenvironment in controlling disease progression, one should initially choose therapies that can be used in the long term, postponing the use of aggressive systemic therapies, such as multidrug chemotherapy, reserved only for rare occasions. In the early stages of the disease, skin-directed therapies constitute the standard of care. In advanced or refractory disease, systemic therapies are used, alone or in combination with skin-directed therapies, despite the rarely observed complete remission rates and the few controlled studies that support them. The role of allo-HSCT is yet to be defined, but it can be useful in some highly selected patients. Treatment availability is also quite heterogeneous, both inside one country and among different countries.^54^ It is expected that the effort made by specialized centers worldwide, with the objective of conducting international multicentric and multidisciplinary studies, will provide a better understanding of the disease, the development of new treatments and a more efficient and uniform standard of care for patients with MF and SS.

## Financial support

None declared.

## Authors’ contributions

José Antonio Sanches: Conception and elaboration of the manuscript.

Jade Cury-Martins: Conception and elaboration of the manuscript.

Rodrigo Martins Abreu: Conception and elaboration of the manuscript.

Denis Miyashiro: Interpretation of data and critical review of the manuscript.

Juliana Pereira: Interpretation of data and critical review of the manuscript.

All authors have approved the final version of the manuscript and agree to be responsible for all aspects of the research, ensuring that issues related to the accuracy or integrity of any part of the study are adequately investigated and resolved.

## Conflicts of interest

Cury-Martins J and Pereira J were sub-investigators in a study funded by Takeda and received fees from Takeda for occasional lectures. Miyashiro D has nothing to declare. Abreu RM is Takeda's scientific medical manager in onco-hematology. Sanches JA was the main investigator of a study funded by Takeda.
